# Supplementary material for: Plasma proteomic signatures of social support and their association with cardiovascular disease and mortality: exploratory analyses in a national cohort study
Source: GeroScience. 2025 Dec 29;48(6):7645–61. doi: 10.1007/s11357-025-02031-8 (PMC13638852; doi:10.1007/s11357-025-02031-8)
Supplement: Supplementary file 1 [file 11357_2025_2031_MOESM1_ESM.docx]

**Supplement**

**eMethods**

**eFigure 1** Participants selection

**eFigure 2** Volcano plots showing proteins associated with social support (a), CVD (b), all-cause mortality (c) in model 2

**eFigure 3** Volcano plots showing the correlation between protein and positive social support in model 1 (a) and in model 2 (b), and relationship strain in model 1 (c) and in model 2 (d).

**eFigure 4** Proteins are shared and distinct for the associations between protein signatures with social support (linear regression analysis), and all-cause mortality (Cox regression) and CVD (Cox regression).

**eFigure 5** **GO enrichment analyses of the 14 proteins associated with social support.**

Rows show the GO terms, the dot sizes show the number of enriched proteins, the colors indicate the FDR-adjusted *P* value and the *x* axis shows the proportion of enriched proteins relative to all proteins associated with the GO term.

**eFigure 6** Venn diagram visualizing the overlap of measured protein levels associated with social support after correction and the proteins associated with social isolation shown in the study by Gong, et al. (2025) and that by Shen, et al. (2025).

**eTable 1** Associations between social support and proteins

**eTable 2** Associations between positive social support, relationship strain and proteins

**eTable 3** Associations between proteins and cardiovascular disease

**eTable 4** Associations between proteins and all-cause mortality

**eTable 5** Association of social support and cardiovascular disease and all-cause mortality

**eTable 6** Association of protein and social support, cardiovascular disease and all-cause mortality

**eTable 7** Sensitivity analyses using complete case analysis for the association of protein and social support, cardiovascular disease and all-cause mortality

**eTable 8** Sensitivity analyses using complete case analysis for the potential protein mediators in the association of social support and cardiovascular disease

**eTable 9** Sensitivity analyses using complete case analysis for exploration of potential protein mediators in the association of social support and all-cause mortality

**eTable 10** Significant functional enrichments in social support-associated proteins

**eReferences**

**eMethods**

**Social support**

Questions on social support were administered via self-completion questionnaire and covered four relationship types: (i) spouse/partner; (ii) children; (iii) extended family members; and (iv) friends. Three items examined the participants’ perception of positive aspects of the social support: (i) “How much do they really understand the way you feel about things?”; (ii) “How much can you rely on them if you have a serious problem?”; and (iii) “How much can you open up to them if you need to talk about your worries?”. These items cover empathy, dependability, and confiding, respectively. The other three items examined negative social support: (i) “How much do they criticize you?”; (ii) “How much do they let you down when you are counting on them?”; and (iii) “How much do they get on your nerves?”. These items cover criticism, being let down, and annoyance, respectively. Responses ranged from “not-at-all” (scored 0) to “a lot” (scored 3). Scores from each relationship were calculated separately using the average of the 3 items (1 or 2 items in the case of item missingness).

Both scores of positive and negative social support were calculated by averaging the score from each relationship, with higher scores showing higher positive and negative social support. When calculating the global score, the items’ scores of negative social supports were first reversed and then a global score was calculated by averaging the 8 scores, with higher scores indicating higher social support. However, given some theoretical overlap between social support and relationship strain, we also split the index into positive social support and negative social strain for sensitivity analyses.

**Covariates**

Directed Acyclic Graph (DAG) was used to identify the potential covariates in the multiple-adjusted models by using the DAGitty online tool. Identified confounders included age, sex, ethnicity, wealth, educational attainment, Body mass index (BMI), and lifestyle behaviours (smoking status, alcohol drinking frequency, and physical activity), assessed through the self-reported questionnaire and respondent interviews. Educational qualifications were categorized into four groups: no educational qualifications; education to GCE/O-levels/national vocational qualification (NVQ) 2 (qualifications at age 16); education to NVQ3/GCE/A-levels (qualifications at age 18); higher qualification/NVQ4/NVQ5/degree). Wealth was recorded using the total net non-pension non-housing wealth (including net value of primary residence, business, and non-housing financial wealth), and participants were categorized into five quantiles. Net non-pension wealth is a robust indicator of socio-economic status in the ELSA population.^1^ Based on the two questions “do you ever smoke” and “are you a current smoker”, smoking status was categorized as never, former and current smoker. Alcohol drinking was dichotomised by the frequency of alcohol consumption into more than 5 days a week or less. Participants were asked about the frequency of vigorous, moderate, and mild physical activity (more than once/week, once/week, 1–3 times/month, or hardly ever). Physical activity was grouped into vigorous (vigorous activity more than once a week), moderate (moderate activity more than once a week), and inactive (mild activity or less frequent activity). BMI was calculated using the participant’s height and weight measured during the nurse visit.

Participants aged >50 years and gave consent for data linkage (n=3,141)

Excluded:

Did not consent the linkage to the death records (n=85)

Age<50 years (n=36)

Participants at wave 4

(n=11,050)

Participants with proteomic data

(n=3,262)

Excluded:

Participants who did not measure the protein (n= 7,868)

Participants in CVD research

(n=2,892)

Excluded:

Participants who had the CVD at baseline (n= 249)

**eFigure 1 Participants selection**

**eFigure 2** **Volcano plots showing proteins associated with social support (a), CVD (b), all-cause mortality (c) in model 2**

The x axis represents beta coefficient in the linear regression (social support and proteins) or hazard ratio in the cox hazard regression model (proteins and CVD and all-cause mortality), and the y axis represents −log10(P values). The blue dashed lines indicate P value <0.05. The red dots indicate the proteins having the FDR adjusted P value < 0.05 and the grey dots indicate the proteins with FDR adjusted P value larger than 0.05. The correlations were all adjusted for age, sex, wealth, education, ethnicity, smoking status, alcohol use, physical activity, and BMI.

**eFigure 3 Volcano plots showing the correlation between protein and positive social support in model 1 (a) and in model 2 (b), and relationship strain in model 1 (c) and in model 2 (d).**

The x axis represents beta coefficient, and the y axis represents −log10(P values). The blue dashed lines indicate P value <0.05. The proteins that marked red indicate the proteins having the FDR adjusted P value < 0.05.

Model 1: adjusted for age, sex, wealth, education, and ethnicity.

Model 2: additionally adjusted for smoking status, alcohol use, physical activity, and BMI.

**eFigure 4** **Proteins are shared and distinct for the associations between protein signatures with social support (linear regression analysis), and all-cause mortality (Cox regression) and CVD (Cox regression).**

The proteins were the significant proteins with FDR adjusted P value less than 0.05 in the minimally adjusted models. The model adjusted for age, sex, education, wealth, and ethnicity.

**eFigure 5 GO enrichment analyses of the 14 proteins associated with social support.**

Rows show the GO terms, the dot sizes show the number of enriched proteins, the colors indicate the FDR-adjusted P value and the x axis shows the proportion of enriched proteins relative to all proteins associated with the GO term.

**eFigure 6 Venn diagram visualizing the overlap of measured protein levels associated with social support after correction and the proteins associated with social isolation shown in the study by Gong, et al. (2025) and that by Shen, et al. (2025).**

The numbers of common and distinct proteins are given, along with the corresponding proteins that are shared with or distinct from those associated with social support.

Shen C, Zhang R, Yu J, Sahakian BJ, Cheng W, Feng J: Plasma proteomic signatures of social isolation and loneliness associated with morbidity and mortality. Nature Human Behaviour 2025, 9(3):569-583.

Gong J, Preminger Z, Steptoe A, Fancourt D: Protein signatures associated with loneliness and social isolation: Plasma proteome analyses in the English Longitudinal Study of Ageing, with causal evidence from Mendelian randomization. Brain Behav Immun 2025, 124:85-94.

**eTable 1 Associations between social support and proteins**

| **Protein** | **Model 1** | | | |  | **Model 2** | | | |
| --- | --- | --- | --- | --- | --- | --- | --- | --- | --- |
|  | Beta | SE | P Value | FDR-adjusted P-value |  | Beta | SE | P Value | FDR-adjusted P-value |
| EFNA4 | -0.146 | 0.045 | 1.154E-03 | **0.031** |  | -0.127 | 0.045 | 4.564E-03 | 0.105 |
| SKR3 | -0.169 | 0.044 | 1.161E-04 | **0.002** |  | -0.127 | 0.043 | 3.317E-03 | 0.101 |
| TN-R | 0.159 | 0.046 | 5.576E-04 | **0.005** |  | 0.085 | 0.045 | 5.530E-02 | 0.313 |
| TNFRSF10A | -0.174 | 0.044 | 8.402E-05 | **0.002** |  | -0.156 | 0.044 | 3.682E-04 | **0.020** |
| TNFRSF11A | -0.185 | 0.044 | 3.087E-05 | **0.018** |  | -0.194 | 0.044 | 1.055E-05 | **0.003** |
| TRAIL-R2 | -0.183 | 0.044 | 2.808E-05 | **0.002** |  | -0.132 | 0.043 | 1.916E-03 | 0.076 |
| Gal-9 | -0.147 | 0.046 | 1.388E-03 | **<0.001** |  | -0.140 | 0.046 | 2.207E-03 | 0.076 |
| FGF-23 | -0.203 | 0.046 | 8.080E-06 | **0.025** |  | -0.182 | 0.045 | 6.409E-05 | **0.004** |
| REN | -0.136 | 0.044 | 1.978E-03 | **0.025** |  | -0.126 | 0.044 | 3.967E-03 | 0.101 |
| VSIG2 | -0.224 | 0.044 | 4.900E-07 | **0.005** |  | -0.180 | 0.044 | 4.668E-05 | **0.004** |
| AMBP | -0.151 | 0.045 | 8.680E-04 | **0.031** |  | -0.150 | 0.045 | 8.591E-04 | **0.040** |
| MMP12 | -0.132 | 0.043 | 2.096E-03 | **0.025** |  | -0.065 | 0.042 | 1.174E-01 | 0.415 |
| ASGR1 | -0.191 | 0.045 | 2.419E-05 | 0.055 |  | -0.187 | 0.044 | 2.344E-05 | **0.003** |
| PSG1 | -0.146 | 0.046 | 1.538E-03 | 0.314 |  | -0.133 | 0.046 | 4.029E-03 | 0.101 |

This table only showed the significant proteins associated with social support in model 1 or model 2 (FDR adjusted P value <0.05).

BMI, body mass index; CVD, cardiovascular disease; CI, confidence interval; HR, hazard ratio.

Model 1: adjusted for age, sex, wealth, education, and ethnicity.

Model 2: additionally adjusted for smoking status, alcohol use, physical activity, and BMI.

**eTable 2 Associations between positive social support, negative relationship strain and proteins**

| **Protein** | **Model 1** | | | |  | **Model 2** | | | |
| --- | --- | --- | --- | --- | --- | --- | --- | --- | --- |
|  | Beta | SE | P Value | FDR-adjusted P-value |  | Beta | SE | P Value | FDR-adjusted P-value |
| **Positive social support** |  |  |  |  |  |  |  |  |  |
| EFNA4 | -0.071 | 0.022 | 1.236E-03 | **0.031** |  | -0.068 | 0.022 | 1.764E-03 | **0.037** |
| SKR3 | -0.088 | 0.021 | 3.420E-05 | **0.002** |  | -0.076 | 0.021 | 3.216E-04 | **0.015** |
| TNFRSF10A | -0.084 | 0.021 | 8.696E-05 | **0.005** |  | -0.086 | 0.021 | 5.467E-05 | **0.005** |
| TNFRSF11A | -0.089 | 0.021 | 3.365E-05 | **0.002** |  | -0.101 | 0.021 | 2.022E-06 | **0.001** |
| TRAIL-R2 | -0.074 | 0.021 | 4.485E-04 | **0.018** |  | -0.060 | 0.021 | 4.071E-03 | 0.061 |
| FGF-23 | -0.094 | 0.022 | 2.446E-05 | **0.002** |  | -0.088 | 0.022 | 7.541E-05 | **0.005** |
| VSIG2 | -0.106 | 0.022 | 1.007E-06 | **<0.001** |  | -0.088 | 0.021 | 3.976E-05 | **0.005** |
| MMP12 | -0.070 | 0.021 | 8.030E-04 | **0.025** |  | -0.055 | 0.021 | 1.088E-02 | 0.09 |
| FGFR2 | -0.075 | 0.022 | 7.609E-04 | **0.025** |  | -0.086 | 0.022 | 1.005E-04 | **0.006** |
| NEFL | -0.075 | 0.019 | 1.028E-04 | **0.005** |  | -0.064 | 0.019 | 8.071E-04 | **0.028** |
| NXPH1 | -0.073 | 0.023 | 1.357E-03 | **0.031** |  | -0.074 | 0.023 | 1.271E-03 | **0.032** |
| PSG1 | -0.074 | 0.022 | 9.221E-04 | **0.025** |  | -0.066 | 0.022 | 3.339E-03 | 0.061 |
| GFR-alpha-1 | -0.063 | 0.021 | 3.000E-03 | 0.055 |  | -0.066 | 0.021 | 1.513E-03 | **0.035** |
| IL12 | -0.035 | 0.022 | 1.180E-01 | 0.314 |  | -0.069 | 0.021 | 1.154E-03 | **0.032** |
| Dkk-4 | -0.053 | 0.022 | 1.400E-02 | 0.104 |  | -0.069 | 0.021 | 1.147E-03 | **0.032** |
| SNCG | -0.051 | 0.022 | 2.400E-02 | 0.133 |  | -0.069 | 0.022 | 1.942E-03 | **0.038** |
| ASGR1 | -0.064 | 0.022 | 4.000E-03 | 0.057 |  | -0.075 | 0.022 | 4.773E-04 | **0.019** |
| **Relationship strain** |  |  |  |  |  |  |  |  |  |
| LEPR | -0.194 | 0.043 | 7.543E-06 | **0.002** |  | -0.166 | 0.043 | 1.045E-04 | **0.029** |

This table only showed the significant proteins associated with social support in model 1 or model 2 (FDR adjusted P value <0.05).

BMI, body mass index; CVD, cardiovascular disease; CI, confidence interval; HR, hazard ratio.

Model 1: adjusted for age, sex, wealth, education, and ethnicity.

Model 2: additionally adjusted for smoking status, alcohol use, physical activity, and BMI.

**eTable 3 Associations between proteins and cardiovascular disease**

| **Protein** | **Model 1** | | | |  | **Model 2** | | | |
| --- | --- | --- | --- | --- | --- | --- | --- | --- | --- |
|  | HR | 95% CI | P Value | FDR-adjusted P-value |  | HR | 95% CI | P Value | FDR-adjusted P-value |
| UNC5C | 1.168 | 1.068,1.276 | 6.700E-04 | **0.008** |  | 1.147 | 1.049,1.254 | 2.780E-03 | **0.045** |
| CLM-6 | 1.128 | 1.037,1.227 | 5.300E-03 | **0.040** |  | 1.089 | 1.000,1.185 | 5.020E-02 | 0.254 |
| EFNA4 | 1.159 | 1.063,1.262 | 8.020E-04 | **0.009** |  | 1.109 | 1.018,1.210 | 1.889E-02 | 0.154 |
| SCARB2 | 1.129 | 1.032,1.235 | 8.614E-03 | **0.049** |  | 1.104 | 1.008,1.210 | 3.264E-02 | 0.200 |
| NCAN | 0.867 | 0.799,0.940 | 5.775E-04 | **0.008** |  | 0.917 | 0.843,0.997 | 4.243E-02 | 0.239 |
| SMPD1 | 1.150 | 1.056,1.252 | 1.316E-03 | **0.013** |  | 1.123 | 1.030,1.224 | 8.820E-03 | 0.101 |
| MSR1 | 1.203 | 1.103,1.312 | 3.39E-05 | **0.001** |  | 1.172 | 1.075,1.279 | 3.700E-04 | **0.013** |
| SIGLEC1 | 1.230 | 1.129,1.34 | 2.822E-06 | **<0.001** |  | 1.189 | 1.090,1.296 | 9.661E-05 | **0.004** |
| BCAN | 0.873 | 0.803,0.949 | 1.585E-03 | **0.015** |  | 0.923 | 0.847,1.007 | 7.122E-02 | 0.277 |
| LAYN | 1.143 | 1.042,1.255 | 4.902E-03 | **0.038** |  | 1.130 | 1.029,1.240 | 1.069E-02 | 0.118 |
| GDF-8 | 0.883 | 0.812,0.96 | 3.719E-03 | **0.031** |  | 0.911 | 0.837,0.992 | 3.211E-02 | 0.200 |
| THY-1 | 1.152 | 1.056,1.258 | 1.600E-03 | **0.015** |  | 1.104 | 1.010,1.207 | 3.026E-02 | 0.199 |
| GFR-alpha-1 | 1.207 | 1.103,1.321 | 4.896E-05 | **0.001** |  | 1.152 | 1.052,1.262 | 2.345E-03 | **0.040** |
| TNFRSF12A | 1.146 | 1.048,1.253 | 2.947E-03 | **0.026** |  | 1.121 | 1.024,1.226 | 1.354E-02 | 0.133 |
| SKR3 | 1.224 | 1.12,1.338 | 1.033E-05 | **<0.001** |  | 1.169 | 1.068,1.279 | 7.629E-04 | **0.021** |
| CPM | 1.159 | 1.067,1.259 | 5.062E-04 | **0.007** |  | 1.105 | 1.015,1.204 | 2.163E-02 | 0.154 |
| CTSC | 1.152 | 1.063,1.249 | 6.535E-04 | **0.008** |  | 1.122 | 1.035,1.216 | 5.330E-03 | 0.072 |
| N2DL-2 | 1.127 | 1.034,1.229 | 6.807E-03 | **0.046** |  | 1.100 | 1.008,1.201 | 3.364E-02 | 0.202 |
| IL12 | 1.117 | 1.029,1.212 | 8.599E-03 | **0.049** |  | 1.122 | 1.030,1.222 | 8.233E-03 | 0.099 |
| EDA2R | 1.294 | 1.157,1.448 | 7.958E-06 | **<0.001** |  | 1.243 | 1.111,1.392 | 1.749E-04 | **0.007** |
| ANGPT1 | 0.893 | 0.823,0.968 | 6.125E-03 | **0.043** |  | 0.905 | 0.835,0.981 | 1.571E-02 | 0.150 |
| TNFRSF10A | 1.263 | 1.158,1.376 | 1.688E-07 | **<0.001** |  | 1.193 | 1.092,1.302 | 9.767E-05 | **0.004** |
| TNFRSF11A | 1.212 | 1.11,1.323 | 2.031E-05 | **<0.001** |  | 1.160 | 1.060,1.268 | 1.242E-03 | **0.029** |
| TRAIL-R2 | 1.207 | 1.106,1.317 | 2.929E-05 | **0.001** |  | 1.124 | 1.027,1.231 | 1.165E-02 | 0.124 |
| IL18 | 1.117 | 1.031,1.211 | 7.102E-03 | **0.046** |  | 1.087 | 1.002,1.179 | 4.441E-02 | 0.241 |
| FGF-21 | 1.122 | 1.033,1.22 | 6.644E-03 | **0.046** |  | 1.065 | 0.978,1.161 | 1.488E-01 | 0.450 |
| FGF-23 | 1.190 | 1.093,1.295 | 6.422E-05 | **0.001** |  | 1.153 | 1.058,1.256 | 1.210E-03 | **0.029** |
| REN | 1.188 | 1.088,1.297 | 1.389E-04 | **0.002** |  | 1.132 | 1.038,1.236 | 5.460E-03 | 0.072 |
| KIM1 | 1.298 | 1.186,1.42 | 2.176E-08 | **<0.001** |  | 1.228 | 1.122,1.345 | 1.100E-05 | **0.001** |
| VSIG2 | 1.192 | 1.093,1.299 | 8.079E-05 | **0.001** |  | 1.147 | 1.051,1.252 | 2.251E-03 | **0.040** |
| AMBP | 1.125 | 1.035,1.223 | 5.981E-03 | **0.043** |  | 1.076 | 0.988,1.171 | 9.243E-02 | 0.323 |
| MMP7 | 1.217 | 1.116,1.327 | 1.064E-05 | **<0.001** |  | 1.163 | 1.066,1.270 | 7.715E-04 | **0.021** |
| PRSS8 | 1.210 | 1.11,1.319 | 1.882E-05 | **<0.001** |  | 1.135 | 1.037,1.242 | 6.265E-03 | 0.079 |
| BNP | 1.283 | 1.173,1.404 | 7.508E-08 | **<0.001** |  | 1.286 | 1.175,1.407 | 7.000E-08 | **<0.001** |
| MMP12 | 1.278 | 1.166,1.4 | 2.025E-07 | **<0.001** |  | 1.22 | 1.110,1.341 | 4.453E-05 | **0.003** |
| ACE2 | 1.271 | 1.167,1.383 | 4.645E-08 | **<0.001** |  | 1.214 | 1.114,1.323 | 1.204E-05 | **0.001** |
| CTSL1 | 1.140 | 1.049,1.238 | 2.198E-03 | **0.020** |  | 1.135 | 1.044,1.235 | 3.164E-03 | **0.049** |
| LEP | 1.229 | 1.118,1.35 | 2.205E-05 | **<0.001** |  | 1.120 | 1.000,1.254 | 4.967E-02 | 0.254 |
| CA5A | 1.148 | 1.058,1.244 | 8.942E-04 | **0.010** |  | 1.102 | 1.015,1.197 | 2.085E-02 | 0.154 |
| HAOX1 | 1.123 | 1.033,1.222 | 6.938E-03 | **0.046** |  | 1.088 | 0.999,1.184 | 5.185E-02 | 0.254 |
| ASGR1 | 1.214 | 1.115,1.321 | 8.932E-06 | **<0.001** |  | 1.135 | 1.039,1.239 | 4.993E-03 | 0.072 |
| CD33 | 1.118 | 1.03,1.213 | 7.608E-03 | **0.047** |  | 1.105 | 1.018,1.199 | 1.709E-02 | 0.154 |
| CDH15 | 1.131 | 1.033,1.238 | 7.853E-03 | **0.047** |  | 1.112 | 1.016,1.217 | 2.174E-02 | 0.154 |
| FGFR2 | 1.128 | 1.038,1.226 | 4.678E-03 | **0.037** |  | 1.081 | 0.994,1.176 | 7.107E-02 | 0.277 |
| IKZF2 | 1.127 | 1.041,1.22 | 3.147E-03 | **0.027** |  | 1.134 | 1.048,1.228 | 1.961E-03 | **0.040** |
| IL3RA | 1.131 | 1.04,1.231 | 4.168E-03 | **0.034** |  | 1.105 | 1.016,1.202 | 2.079E-02 | 0.154 |
| PAEP | 1.125 | 1.031,1.227 | 8.092E-03 | **0.048** |  | 1.111 | 1.018,1.212 | 1.913E-02 | 0.154 |
| RPS6KB1 | 1.144 | 1.055,1.241 | 1.241E-03 | **0.013** |  | 1.135 | 1.047,1.232 | 2.280E-03 | **0.040** |
| SFRP1 | 1.128 | 1.033,1.233 | 7.511E-03 | **0.047** |  | 1.091 | 0.999,1.193 | 5.408E-02 | 0.254 |

BMI, body mass index; CVD, cardiovascular disease; CI, confidence interval; HR, hazard ratio.

Model 1: adjusted for age, sex, wealth, education, and ethnicity.

Model 2: additionally adjusted for smoking status, alcohol use, physical activity, and BMI.

**eTable 4 Associations between proteins and all-cause mortality**

| **Protein** | **Model 1** | | | |  | **Model 2** | | | |
| --- | --- | --- | --- | --- | --- | --- | --- | --- | --- |
|  | HR | 95% CI | P Value | FDR-adjusted P-value |  | HR | 95% CI | P Value | FDR-adjusted P-value |
| UNC5C | 1.104 | 1.026,1.188 | 7.951E-03 | **0.034** |  | 1.118 | 1.039,1.203 | 2.801E-03 | **0.020** |
| VWC2 | 1.107 | 1.029,1.190 | 6.290E-03 | **0.029** |  | 1.095 | 1.018,1.177 | 1.433E-02 | 0.069 |
| CLM-6 | 1.102 | 1.028,1.182 | 6.586E-03 | **0.030** |  | 1.074 | 1.001,1.153 | 4.617E-02 | 0.170 |
| NBL1 | 0.905 | 0.847,0.968 | 3.573E-03 | **0.018** |  | 0.921 | 0.861,0.985 | 1.695E-02 | 0.079 |
| EFNA4 | 1.174 | 1.094,1.260 | 9.150E-06 | **<0.001** |  | 1.132 | 1.054,1.215 | 6.538E-04 | **0.007** |
| SCARB2 | 1.142 | 1.062,1.228 | 3.694E-04 | **0.002** |  | 1.141 | 1.061,1.226 | 3.898E-04 | **0.004** |
| NCAN | 0.850 | 0.794,0.911 | 4.343E-06 | **<0.001** |  | 0.890 | 0.830,0.954 | 9.868E-04 | **0.010** |
| SMPD1 | 1.167 | 1.085,1.256 | 3.894E-05 | **<0.001** |  | 1.104 | 1.025,1.189 | 9.185E-03 | **0.046** |
| MSR1 | 1.124 | 1.044,1.211 | 1.902E-03 | **0.010** |  | 1.123 | 1.043,1.209 | 2.185E-03 | **0.016** |
| SIGLEC1 | 1.167 | 1.086,1.253 | 2.815E-05 | **<0.001** |  | 1.158 | 1.078,1.245 | 6.512E-05 | **0.001** |
| CNTN5 | 0.869 | 0.809,0.932 | 1.048E-04 | **0.001** |  | 0.890 | 0.827,0.957 | 1.747E-03 | **0.014** |
| CLEC1B | 0.921 | 0.863,0.982 | 1.236E-02 | **0.049** |  | 0.912 | 0.855,0.974 | 5.898E-03 | **0.031** |
| BCAN | 0.809 | 0.754,0.869 | 6.817E-09 | **<0.001** |  | 0.855 | 0.795,0.918 | 2.007E-05 | **<0.001** |
| LAYN | 1.202 | 1.113,1.297 | 2.917E-06 | **<0.001** |  | 1.174 | 1.087,1.267 | 4.663E-05 | **0.001** |
| GDF-8 | 0.844 | 0.786,0.906 | 3.655E-06 | **<0.001** |  | 0.902 | 0.839,0.969 | 5.075E-03 | **0.030** |
| WFIKKN1 | 0.869 | 0.809,0.934 | 1.344E-04 | **0.001** |  | 0.927 | 0.862,0.998 | 4.343E-02 | 0.167 |
| GFR-alpha-1 | 1.187 | 1.100,1.281 | 1.125E-05 | **<0.001** |  | 1.157 | 1.072,1.249 | 1.844E-04 | **0.002** |
| NTRK2 | 0.901 | 0.840,0.966 | 3.412E-03 | **0.017** |  | 0.924 | 0.861,0.991 | 2.753E-02 | 0.117 |
| SCARF2 | 1.112 | 1.028,1.204 | 8.627E-03 | **0.037** |  | 1.114 | 1.029,1.206 | 7.901E-03 | **0.040** |
| TNFRSF12A | 1.175 | 1.091,1.265 | 2.172E-05 | **<0.001** |  | 1.161 | 1.078,1.251 | 9.278E-05 | **0.001** |
| SKR3 | 1.219 | 1.133,1.312 | 1.472E-07 | **<0.001** |  | 1.145 | 1.063,1.233 | 3.549E-04 | **0.004** |
| GCP5 | 0.881 | 0.824,0.942 | 2.265E-04 | **0.002** |  | 0.901 | 0.843,0.964 | 2.531E-03 | **0.018** |
| N2DL-2 | 1.098 | 1.022,1.179 | 1.068E-02 | **0.044** |  | 1.071 | 0.996,1.151 | 6.420E-02 | 0.211 |
| CLM-1 | 1.104 | 1.031,1.181 | 4.430E-03 | **0.021** |  | 1.068 | 0.998,1.143 | 5.734E-02 | 0.195 |
| SPOCK1 | 0.884 | 0.826,0.947 | 4.299E-04 | **0.003** |  | 0.933 | 0.871,1.000 | 4.959E-02 | 0.178 |
| EDA2R | 1.393 | 1.268,1.531 | 1.153E-11 | **<0.001** |  | 1.362 | 1.239,1.496 | 2.228E-10 | **<0.001** |
| NTRK3 | 0.916 | 0.856,0.980 | 1.118E-02 | **0.045** |  | 0.949 | 0.886,1.016 | 1.306E-01 | 0.343 |
| TN-R | 0.807 | 0.752,0.866 | 3.787E-09 | **<0.001** |  | 0.873 | 0.812,0.938 | 2.304E-04 | **0.003** |
| ANGPT1 | 0.903 | 0.846,0.965 | 2.599E-03 | **0.014** |  | 0.908 | 0.851,0.969 | 3.924E-03 | 0.025 |
| SLAMF7 | 1.158 | 1.082,1.239 | 2.361E-05 | **<0.001** |  | 1.153 | 1.078,1.233 | 3.720E-05 | **0.001** |
| PGF | 1.201 | 1.115,1.294 | 1.581E-06 | **<0.001** |  | 1.174 | 1.089,1.266 | 3.118E-05 | **0.001** |
| IL-4RA | 1.144 | 1.067,1.226 | 1.694E-04 | **0.001** |  | 1.104 | 1.03,1.183 | 5.321E-03 | **0.031** |
| TNFRSF10A | 1.292 | 1.200,1.392 | 1.898E-11 | **<0.001** |  | 1.231 | 1.142,1.327 | 7.641E-08 | **<0.001** |
| TNFRSF11A | 1.199 | 1.116,1.288 | 9.054E-07 | **<0.001** |  | 1.183 | 1.101,1.272 | 5.695E-06 | **<0.001** |
| PAR-1 | 1.116 | 1.043,1.193 | 1.430E-03 | **0.008** |  | 1.099 | 1.028,1.175 | 5.738E-03 | **0.031** |
| TRAIL-R2 | 1.372 | 1.273,1.479 | 5.356E-16 | **<0.001** |  | 1.262 | 1.169,1.363 | 4.109E-09 | **<0.001** |
| IL1RL2 | 0.895 | 0.834,0.960 | 2.037E-03 | **0.011** |  | 0.926 | 0.863,0.994 | 3.329E-02 | 0.135 |
| Gal-9 | 1.157 | 1.078,1.242 | 5.665E-05 | **0.001** |  | 1.120 | 1.043,1.203 | 1.869E-03 | **0.015** |
| IL18 | 1.155 | 1.079,1.236 | 3.760E-05 | **<0.001** |  | 1.128 | 1.054,1.208 | 5.540E-04 | **0.006** |
| FGF-21 | 1.136 | 1.058,1.221 | 4.783E-04 | **0.003** |  | 1.091 | 1.015,1.172 | 1.809E-02 | 0.083 |
| PIgR | 1.103 | 1.031,1.181 | 4.400E-03 | **0.021** |  | 1.042 | 0.974,1.116 | 2.325E-01 | 0.490 |
| FGF-23 | 1.202 | 1.121,1.289 | 3.130E-07 | **<0.001** |  | 1.173 | 1.094,1.258 | 8.628E-06 | **<0.001** |
| SPON2 | 1.145 | 1.067,1.228 | 1.787E-04 | **0.001** |  | 1.128 | 1.051,1.211 | 9.350E-04 | **0.010** |
| REN | 1.207 | 1.121,1.299 | 6.495E-07 | **<0.001** |  | 1.179 | 1.094,1.269 | 1.592E-05 | **<0.001** |
| KIM1 | 1.397 | 1.298,1.503 | 2.784E-18 | **<0.001** |  | 1.323 | 1.228,1.425 | 3.668E-13 | **<0.001** |
| THBS2 | 1.119 | 1.044,1.199 | 1.495E-03 | **0.008** |  | 1.128 | 1.05,1.212 | 1.064E-03 | **0.010** |
| VSIG2 | 1.220 | 1.138,1.308 | 2.725E-08 | **<0.001** |  | 1.162 | 1.084,1.246 | 2.700E-05 | **0.001** |
| AMBP | 1.135 | 1.059,1.218 | 3.958E-04 | **0.003** |  | 1.103 | 1.028,1.184 | 6.724E-03 | **0.035** |
| CCL17 | 1.152 | 1.076,1.234 | 5.661E-05 | **0.001** |  | 1.109 | 1.035,1.187 | 3.293E-03 | **0.023** |
| MMP7 | 1.325 | 1.230,1.427 | 2.842E-13 | **<0.001** |  | 1.258 | 1.167,1.357 | 3.442E-09 | **<0.001** |
| PRSS8 | 1.275 | 1.185,1.372 | 1.539E-10 | **<0.001** |  | 1.186 | 1.100,1.279 | 1.107E-05 | **<0.001** |
| AGRP | 1.122 | 1.051,1.198 | 5.870E-04 | **0.004** |  | 1.102 | 1.032,1.176 | 3.693E-03 | **0.024** |
| MARCO | 1.097 | 1.025,1.173 | 7.426E-03 | **0.033** |  | 1.117 | 1.043,1.195 | 1.540E-03 | **0.014** |
| BNP | 1.163 | 1.082,1.249 | 4.406E-05 | **<0.001** |  | 1.148 | 1.068,1.233 | 1.732E-04 | **0.002** |
| MMP12 | 1.376 | 1.274,1.487 | 1.770E-15 | **<0.001** |  | 1.264 | 1.168,1.368 | 8.982e-09 | **<0.001** |
| ACE2 | 1.157 | 1.075,1.244 | 9.733E-05 | **0.001** |  | 1.128 | 1.047,1.216 | 1.689E-03 | **0.014** |
| CTSL1 | 1.150 | 1.072,1.232 | 9.349E-05 | **0.001** |  | 1.145 | 1.068,1.228 | 1.532E-04 | **0.002** |
| hOSCAR | 1.152 | 1.075,1.234 | 6.879E-05 | **0.001** |  | 1.104 | 1.029,1.184 | 5.736E-03 | **0.031** |
| TNFRSF13B | 1.140 | 1.064,1.222 | 2.193E-04 | **0.002** |  | 1.103 | 1.029,1.182 | 5.472E-03 | **0.031** |
| CD4 | 1.138 | 1.062,1.220 | 2.650E-04 | **0.002** |  | 1.104 | 1.031,1.183 | 4.769E-03 | **0.029** |
| ASGR1 | 1.165 | 1.086,1.250 | 2.380E-05 | **<0.001** |  | 1.121 | 1.043,1.205 | 2.007E-03 | **0.015** |
| DEFB4A | 1.098 | 1.025,1.177 | 7.799E-03 | **0.034** |  | 1.061 | 0.989,1.137 | 9.854E-02 | 0.283 |
| GGT5 | 1.096 | 1.023,1.174 | 9.546E-03 | **0.040** |  | 1.090 | 1.017,1.168 | 1.505E-02 | 0.072 |
| KIR2DL3 | 1.121 | 1.048,1.199 | 9.693E-04 | **0.006** |  | 1.107 | 1.034,1.185 | 3.569E-03 | **0.024** |
| LTBP3 | 1.130 | 1.052,1.215 | 9.006E-04 | **0.005** |  | 1.125 | 1.046,1.210 | 1.628E-03 | **0.014** |
| NEFL | 1.358 | 1.243,1.483 | 1.914E-11 | **<0.001** |  | 1.340 | 1.227,1.464 | 1.501e-10 | **<0.001** |
| PLA2G10 | 1.110 | 1.032,1.193 | 5.014E-03 | **0.023** |  | 1.108 | 1.031,1.191 | 5.569E-03 | **0.031** |
| PSG1 | 1.093 | 1.019,1.172 | 1.259E-02 | **0.050** |  | 1.079 | 1.006,1.158 | 3.338E-02 | 0.135 |
| SFRP1 | 1.169 | 1.083,1.261 | 6.107E-05 | **0.001** |  | 1.135 | 1.052,1.225 | 1.090E-03 | **0.010** |
| SNCG | 1.117 | 1.039,1.200 | 2.711E-03 | **0.014** |  | 1.112 | 1.034,1.196 | 4.405E-03 | **0.028** |

BMI, body mass index; CI, confidence interval; HR, hazard ratio.

Model 1: adjusted for age, sex, wealth, education, and ethnicity.

Model 2: additionally adjusted for smoking status, alcohol use, physical activity, and BMI

**eTable 5 Association of social support and cardiovascular disease and all-cause mortality**

|  | **Model 1** | |  | **Model 2** | |  |
| --- | --- | --- | --- | --- | --- | --- |
|  | **HR** | **95% CI** |  | **HR** | **95% CI** |  |
| **CVD** |  |  |  |  |  |  |
| Social support | 0.723 | 0.588,0.888 |  | 0.747 | 0.609,0.918 | |
| Positive social support | 0.878 | 0.797,0.966 |  | 0.878 | 0.798,0.967 | |
| Relationship strain | 1.047 | 0.868,1.263 |  | 0.993 | 0.823,1.199 | |
|  |  |  | |  |  | |
| **All-cause mortality** |  |  | |  |  | |
| Social support | 0.654 | 0.548,0.780 | | 0.718 | 0.602,0.857 | |
| Positive social support | 0.823 | 0.759,0.892 | | 0.855 | 0.788,0.927 | |
| Relationship strain | 0.955 | 0.807,1.129 | | 0.936 | 0.791,1.108 | |

BMI, body mass index; CVD, cardiovascular disease; CI, confidence interval; HR, hazard ratio.

Model 1: adjusted for age, sex, wealth, education, and ethnicity.

Model 2: additionally adjusted for smoking status, alcohol use, physical activity, and BMI.

**eTable 6 Association of protein and social support, cardiovascular disease and all-cause mortality**

|  | **Social support** | | | |  | **CVD** | | | |  | **All-cause mortality** | | | |
| --- | --- | --- | --- | --- | --- | --- | --- | --- | --- | --- | --- | --- | --- | --- |
| Protein | Beta | SE | P Value | FDR-adjusted P-value |  | HR | 95% CI | P value | FDR-adjusted P-value |  | HR | 95% CI | P value | FDR-adjusted P-value |
| EFNA4 | -0.146 | 0.045 | 1.154E-03 | **0.032** |  | 1.159 | 1.063, 1.262 | 8.020E-04 | **<0.001** |  | 1.107 | 1.029, 1.190 | 9.150E-06 | **0.0289** |
| SKR3 | -0.169 | 0.044 | 1.161E-04 | **0.005** |  | 1.224 | 1.120, 1.338 | 1.033E-05 | **<0.001** |  | 1.219 | 1.133, 1.312 | 1.472E-07 | **3.386e-06** |
| TN-R | 0.159 | 0.046 | 5.576E-04 | **0.019** |  | 0.957 | 0.880, 1.039 | 2.946E-01 | 0.521 |  | 0.807 | 0.752, 0.866 | 4.282E-10 | **1.161e-07** |
| TNFRSF10A | -0.174 | 0.044 | 8.402E-05 | **0.004** |  | 1.263 | 1.158, 1.376 | 1.700E-07 | **<0.001** |  | 1.292 | 1.200, 1.392 | 9.596E-13 | **7.546e-10** |
| TNFRSF11A | -0.185 | 0.044 | 3.087E-05 | **0.002** |  | 1.212 | 1.11, 1.323 | 2.031E-05 | **<0.001** |  | 1.199 | 1.116, 1.288 | 9.054E-07 | **1.666e-05** |
| TRAIL-R2 | -0.183 | 0.044 | 2.808E-05 | **0.002** |  | 1.207 | 1.106, 1.317 | 2.929E-05 | **<0.001** |  | 1.372 | 1.273, 1.479 | 1.373E-17 | **7.391e-14** |
| Gal-9 | -0.147 | 0.046 | 1.388E-03 | **0.035** |  | 1.105 | 1.018, 1.199 | 1.754E-02 | 0.0793 |  | 1.157 | 1.078, 1.242 | 5.665E-05 | **0.0005** |
| FGF-23 | -0.203 | 0.046 | 8.080E-06 | **0.001** |  | 1.190 | 1.093, 1.295 | 6.422E-05 | **0.0010** |  | 1.202 | 1.121, 1.289 | 3.130E-07 | **6.646e-06** |
| REN | -0.136 | 0.044 | 1.978E-03 | **0.041** |  | 1.188 | 1.088, 1.297 | 1.389E-04 | **0.0020** |  | 1.207 | 1.121, 1.299 | 6.495E-07 | **1.280e-05** |
| VSIG2 | -0.224 | 0.044 | 4.900E-07 | **<0.001** |  | 1.192 | 1.093, 1.299 | 8.079E-05 | **0.0012** |  | 1.22 | 1.138, 1.308 | 2.725E-08 | **6.837e-07** |
| AMBP | -0.151 | 0.045 | 8.680E-04 | **0.027** |  | 1.125 | 1.035, 1.223 | 5.981E-03 | **0.0433** |  | 1.135 | 1.059, 1.218 | 3.958E-04 | **0.002541** |
| MMP12 | -0.132 | 0.043 | 2.096E-03 | **0.041** |  | 1.278 | 1.166, 1.400 | 2.000E-07 | **<0.001** |  | 1.376 | 1.274, 1.487 | 1.770E-15 | **1.628e-13** |
| ASGR1 | -0.191 | 0.045 | 2.419E-05 | **0.002** |  | 1.214 | 1.115, 1.321 | 8.930E-06 | **0.0002** |  | 1.165 | 1.086, 1.250 | 2.380E-05 | **0.0002** |
| PSG1 | -0.146 | 0.046 | 1.538E-03 | **0.035** |  | 1.082 | 0.995, 1.176 | 6.699E-02 | 0.2054 |  | 1.093 | 1.019, 1.172 | 1.259E-02 | **0.0498** |

This table only showed the significant proteins associated with social support (FDR adjusted P value <0.05), and the association with CVD and all-cause mortality

Model adjusted for age, sex, wealth, education, and ethnicity.

**eTable 7 Sensitivity analyses using complete case analysis for the association of protein and social support, cardiovascular disease and all-cause mortality**

|  | **Social support** | | | |  | **CVD** | | | |  | **All-cause mortality** | | | |
| --- | --- | --- | --- | --- | --- | --- | --- | --- | --- | --- | --- | --- | --- | --- |
| Protein | Beta | SE | P Value | FDR-adjusted P-value |  | HR | 95% CI | P value | FDR-adjusted P-value |  | HR | 95% CI | P value | FDR-adjusted P-value |
| EFNA4 | -0.153 | 0.046 | 8.989E-04 | **0.027** |  | 1.172 | 1.074,1.28 | 3.803E-04 | **0.005** |  | 1.17 | 1.089,1.258 | 1.919E-05 | **<0.001** |
| SKR3 | -0.178 | 0.045 | 8.152E-05 | **0.005** |  | 1.246 | 1.138,1.364 | 1.892E-06 | **<0.001** |  | 1.218 | 1.129,1.313 | 2.810E-07 | **<0.001** |
| TN-R | 0.173 | 0.047 | 2.289E-04 | **0.008** |  | 0.948 | 0.872,1.032 | 2.159E-01 | 0.403 |  | 0.796 | 0.741,0.855 | 4.282E-10 | **<0.001** |
| TNFRSF10A | -0.168 | 0.045 | 2.064E-04 | **0.008** |  | 1.283 | 1.175,1.401 | 2.937E-08 | **<0.001** |  | 1.314 | 1.219,1.416 | 9.596E-13 | **<0.001** |
| TNFRSF11A | -0.174 | 0.046 | 1.385E-04 | **0.006** |  | 1.225 | 1.121,1.339 | 8.254E-06 | **<0.001** |  | 1.210 | 1.125,1.302 | 3.281E-07 | **<0.001** |
| TRAIL-R2 | -0.174 | 0.045 | 9.429E-05 | **0.005** |  | 1.212 | 1.108,1.325 | 2.559E-05 | **<0.001** |  | 1.394 | 1.292,1.505 | 1.373E-17 | **<0.001** |
| FGF-23 | -0.191 | 0.046 | 3.541E-05 | **0.003** |  | 1.202 | 1.103,1.31 | 2.711E-05 | **<0.001** |  | 1.204 | 1.121,1.293 | 3.219E-07 | **<0.001** |
| REN | -0.139 | 0.045 | 1.847E-03 | **0.036** |  | 1.193 | 1.0901.305 | 1.245E-04 | **0.002** |  | 1.218 | 1.131,1.311 | 1.813E-07 | **<0.001** |
| VSIG2 | -0.233 | 0.045 | 3.181E-07 | **<0.001** |  | 1.195 | 1.094,1.306 | 8.062E-05 | **0.001** |  | 1.225 | 1.141,1.315 | 2.130E-08 | **<0.001** |
| AMBP | -0.146 | 0.046 | 1.649E-03 | **0.036** |  | 1.13 | 1.038,1.231 | 4.812E-03 | **0.030** |  | 1.15 | 1.071,1.235 | 1.156E-04 | **0.001** |
| MMP12 | -0.137 | 0.044 | 1.754E-03 | **0.036** |  | 1.286 | 1.173,1.411 | 8.194E-08 | **<0.001** |  | 1.395 | 1.29,1.509 | 8.436E-17 | **<0.001** |
| ASGR1 | -0.156 | 0.047 | 9.943E-04 | **0.027** |  | 1.224 | 1.122,1.335 | 5.286E-06 | **<0.001** |  | 1.173 | 1.091,1.261 | 1.480E-05 | **<0.001** |
| PSG1 | -0.211 | 0.047 | 6.865E-06 | **0.001** |  | 1.071 | 0.984,1.165 | 1.139E-01 | 0.270 |  | 1.087 | 1.013,1.166 | 2.109E-02 | 0.077 |
| Gal-9 | -0.134 | 0.047 | 1.444E-03 | 0.072 |  | 1.100 | 1.011,1.196 | 2.609E-02 | 0.099 |  | 1.160 | 1.079,1.246 | 5.236E-05 | **<0.001** |

BMI, body mass index; CVD, cardiovascular disease; CI, confidence interval; HR, hazard ratio; SE, Standard Error.

This table only shows the significant proteins associated with social support (FDR adjusted P value <0.05) except for Gal-9 which has been found to be associated with social support in the main analysis.

All models adjusted for age, sex, wealth, education, and ethnicity

**eTable 8 Sensitivity analyses using complete case analysis for the potential protein mediators in the association of social support and cardiovascular disease**

| **Outcome** | **Total effect** |  | **Natural Direct effect** |  | **Natural Indirect effect** | **Proportion mediated, % (95% CI)** |
| --- | --- | --- | --- | --- | --- | --- |
|  | **HR (95% CI)** |  | **HR (95% CI)** |  | **HR (95% CI)** |  |
| **Single mediator** |  |  |  |  |  |  |
| EFNA4 | 0.732(0.595,0.910) |  | 0.749(0.607,0.936) |  | 0.978(0.956,0.993) | 6.2 (1.44, 28.0) |
| SKR3 | 0.731(0.584,0.904) |  | 0.758(0.612,0.939) |  | 0.964(0.938,0.983) | 10.0 (3.5, 30.5) |
| TNFRSF10A | 0.750(0.617,0.938) |  | 0.781(0.644,0.981) |  | 0.961(0.934,0.981) | 12.2 (5.1, 66.5) |
| TNFRSF11A | 0.750(0.616,0.916) |  | 0.775(0.641,0.944) |  | 0.967(0.939,0.986) | 10.3 (3.8, 41.1) |
| TRAIL-R2 | 0.753(0.607,0.906) |  | 0.777(0.628,0.934) |  | 0.969(0.947,0.986) | 9.6 (3.2, 32.5) |
| FGF-23 | 0.754(0.606,0.953) |  | 0.779(0.630,0.975) |  | 0.968(0.942,0.989) | 10.0 (2.7, 51.8) |
| REN | 0.749(0.601,0.924) |  | 0.766(0.615,0.951) |  | 0.978(0.958,0.995) | 6.7 (1.1, 28.6) |
| VSIG2 | 0.746(0.611,0.907) |  | 0.778(0.628,0.938) |  | 0.959(0.936,0.982) | 12.5 (3.8, 40.1) |
| AMBP | 0.751(0.634,0.904) |  | 0.764(0.656,0.925) |  | 0.982(0.963,0.996) | 5.4 (0.9, 21.1) |
| MMP12 | 0.747(0.628,0.911) |  | 0.773(0.653,0.941) |  | 0.967(0.933,0.988) | 10.2 (3.5, 33.4) |
| ASGR1 | 0.719(0.594,0.880) |  | 0.748(0.619,0.907) |  | 0.961(0.931,0.985) | 10.5 (3.8, 32.1) |
| **Multiple mediators** |  |  |  |  |  |  |
| Total mediation | 0.707(0.573,0.887) |  | 0.782(0.631,0.981) |  | 0.904(0.854,0.946) | 25.6 (11.0, 83.2) |

All the significant mediators in the single mediation model were put in one model with the multiple mediators.

**eTable 9 Sensitivity analyses using complete case analysis for exploration of potential protein mediators in the association of social support and all-cause mortality**

| **Outcome** | **Total effect** |  | **Natural Direct effect** |  | **Natural Indirect effect** | **Proportion mediated, % (95% CI)** |
| --- | --- | --- | --- | --- | --- | --- |
|  | **HR (95% CI)** |  | **HR (95% CI)** |  | **HR (95% CI)** |  |
| **Single mediator** |  |  |  |  |  |  |
| EFNA4 | 0.650(0.512,0.776) |  | 0.665(0.520,0.793) |  | 0.978(0.955,0.991) | 4.3 (1.4, 11.3) |
| SKR3 | 0.648(0.530,0.783) |  | 0.670(0.548,0.803) |  | 0.967(0.945,0.985) | 6.2 (2.3, 14.5) |
| TN-R | 0.661(0.535,0.761) |  | 0.686(0.555,0.789) |  | 0.964(0.939,0.983) | 7.3 (2.5, 15.5) |
| TNFRSF10A | 0.651(0.543,0.781) |  | 0.681(0.570,0.819) |  | 0.956(0.931,0.981) | 8.5 (3.7, 18.1) |
| TNFRSF11A | 0.652(0.531,0.766) |  | 0.673(0.551,0.782) |  | 0.969(0.946,0.986) | 6.1 (1.9, 11.3) |
| TRAIL-R2 | 0.650(0.542,0.797) |  | 0.687(0.570,0.861) |  | 0.945(0.915,0.970) | 10.7 (4.9, 27.7) |
| FGF-23 | 0.655(0.536,0.793) |  | 0.677(0.556,0.817) |  | 0.967(0.942,0.984) | 6.5 (2.3, 15.2) |
| REN | 0.657(0.532,0.781) |  | 0.675(0.546,0.807) |  | 0.974(0.952,0.992) | 5.1 (1.5, 11.4) |
| VSIG2 | 0.650(0.548,0.793) |  | 0.680(0.578,0.825) |  | 0.956(0.933,0.978) | 8.5 (3.7, 17.8) |
| AMBP | 0.654(0.530,0.776) |  | 0.667(0.545,0.796) |  | 0.981(0.963,0.994) | 3.7 (1.3, 9.5) |
| MMP12 | 0.644(0.535,0.772) |  | 0.673(0.561,0.809) |  | 0.956(0.931,0.983) | 8.3 (3.0, 17.0) |
| ASGR1 | 0.653(0.529,0.766) |  | 0.673(0.546,0.781) |  | 0.969(0.950,0.988) | 5.9 (2.0,11.2) |
|  |  |  |  |  |  |  |
| **Multiple mediators** |  |  |  |  |  |  |
| **Total mediation** | 0.644(0.506,0.767) |  | 0.749(0.607,0.894) |  | 0.86(0.799,0.905) | 29.4 (17.2, 57.2) |

**eTable 10. Significant functional enrichments in social support-associated proteins**

| **Term id** | **Source** | **Term name** | **FDR-adjusted P-value** | **Intersection size** |
| --- | --- | --- | --- | --- |
| GO:0005035 | GO:MF | death receptor activity | 0.0007 | 2 |
| GO:0046904 | GO:MF | calcium oxalate binding | 0.0208 | 1 |
| GO:0004873 | GO:MF | asialoglycoprotein receptor activity | 0.0208 | 1 |
| GO:0019862 | GO:MF | IgA binding | 0.0234 | 1 |
| GO:0045569 | GO:MF | TRAIL binding | 0.0312 | 1 |
| GO:0019855 | GO:MF | calcium channel inhibitor activity | 0.0403 | 1 |
| GO:0005031 | GO:MF | tumor necrosis factor receptor activity | 0.0403 | 1 |
| GO:0042806 | GO:MF | fucose binding | 0.0403 | 1 |
| GO:0030246 | GO:MF | carbohydrate binding | 0.0403 | 2 |
| GO:0005005 | GO:MF | transmembrane-ephrin receptor activity | 0.0436 | 1 |
| GO:0005159 | GO:MF | insulin-like growth factor receptor binding | 0.0453 | 1 |
| GO:0046875 | GO:MF | ephrin receptor binding | 0.0458 | 1 |
| GO:0004175 | GO:MF | endopeptidase activity | 0.0458 | 2 |
| GO:0005537 | GO:MF | D-mannose binding | 0.0458 | 1 |
| GO:0005003 | GO:MF | ephrin receptor activity | 0.0458 | 1 |
| GO:0004888 | GO:MF | transmembrane signaling receptor activity | 0.0458 | 3 |
| GO:0004190 | GO:MF | aspartic-type endopeptidase activity | 0.0458 | 1 |
| GO:0019865 | GO:MF | immunoglobulin binding | 0.0458 | 1 |
| GO:0070001 | GO:MF | aspartic-type peptidase activity | 0.0458 | 1 |
| GO:0060089 | GO:MF | molecular transducer activity | 0.0481 | 3 |
| GO:0038023 | GO:MF | signaling receptor activity | 0.0481 | 3 |

This table shows the significantly enriched pathways of the 14 proteins associated with social support (FDR-adjusted P-value <0.05).

**eReferences**

1.	Banks J, Karlsen S, Oldfield Z. Socio-economic position. Institute for Fiscal Studies; 2003.
